# Network meta-analysis of intravitreal conbercept as an adjuvant to vitrectomy for proliferative diabetic retinopathy

**DOI:** 10.3389/fendo.2023.1098165

**Published:** 2023-02-22

**Authors:** Weiwei Wang, Chaoyi Qu, Huanhuan Yan

**Affiliations:** Shaanxi Eye Hospital, Xi’an People’s Hospital (Xi’an Fourth Hospital), Xi’an, China

**Keywords:** proliferative diabetic retinopathy, vitrectomy, conbercept, intravitreal, network meta-analysis

## Abstract

**Purpose:**

Intravitreal Conbercept (IVC) has been shown to be effective in treating proliferative diabetic retinopathy (PDR) as an adjuvant in pars plana vitrectomy (PPV); however, the best timing of IVC injection remains unknown. This network meta-analysis (NMA) sought to ascertain the comparative efficacy of different timings of IVC injection as an adjuvant to PPV on PDR.

**Methods:**

A comprehensive literature search was conducted in PubMed, EMBASE, and the Cochrane Library to identify relevant studies published before August 11, 2022. According to the mean time of IVC injection before PPV, the strategy was defined as very long interval if it was > 7 days but ≤ 9 days, long interval if it was > 5 days but ≤ 7 days, mid interval if it was > 3 days but ≤ 5 days, and short interval if it was ≤ 3 days, respectively. The strategy was defined as perioperative IVC if IVC was injected both before and at the end of PPV, and the strategy was intraoperative IVC if injected immediately at the end of PPV. The mean difference (MD) and odds ratio (OR) with corresponding 95% confidence interval (CI) for continuous and binary variables, respectively, were computed through network meta-analysis using Stata 14.0 MP.

**Results:**

Eighteen studies involving 1149 patients were included. There was no statistical difference between intraoperative IVC and control in treating PDR. Except for a very long interval, preoperative IVC significantly shortened operation time, and reduced intraoperative bleeding and iatrogenic retinal breaks. Long and short intervals reduced endodiathermy application, and mid and short intervals reduced postoperative vitreous hemorrhage. Moreover, long and mid intervals improved BCVA and central macular thickness. However, very long interval was associated with an increased risk of postoperative vitreous hemorrhage (RR: 3.27, 95%CI: 1.84 to 5.83). Moreover, mid interval was better than intraoperative IVC in shortening operation time (MD: -19.74, 95%CI: -33.31 to -6.17).

**Conclusions:**

There are no discernible effects of intraoperative IVC on PDR, but preoperative IVC, except for very long interval, is an effective adjuvant to PPV for treating PDR.

## Introduction

Diabetic retinopathy (DR), the most common diabetic complication, is characterized by damage and abnormalities in retinal blood vessels, which can result in visual impairment and blindness (1). Depending on the severity, DR can be classified into three subtypes: non-proliferative DR, proliferative DR (PDR), and diabetic macular edema (2). PDR is one of the most common causes of blindness in DR patients and is linked to vitreous hemorrhage, traction detachment, and neovascular glaucoma (3–5). DR affected approximately 103 million adults worldwide in 2020, which is expected to reach 160 million by 2045 (6). Therefore, it is critical to treat patients with PDR effectively.

Panretinal photocoagulation (5) and vitrectomy (7) are two traditional treatment options for PDR. Pars plana vitrectomy (PPV) remains the preferred treatment for PDR (8), as it removes long-standing hematoma in the vitreous cavity, blocks the pathways to neovascularization, and restores the stable intraocular structure to the retina (9). However, this procedure may be associated with an increased risk of several complications, such as retinal detachment (RD) and repeated vitreous hemorrhage. These complications can undoubtedly delay patients’ vision recovery and increase surgical costs (10). Clinical practitioners are trying to mitigate the possible negative effects of PPV by different approaches.

Vascular endothelial growth factor (VEGF) plays a central role in the development of PDR (11), and it has been demonstrated that intravitreal anti-VEGF decreases the need for repeated vitrectomy and recurrent vitreous hemorrhage (12, 13). As a novel anti-VEGF drug, Conbercept was approved by the China Food and Drug Administration (CFDA) to treat age-related macular degeneration in 2013 (14). Conbercept is a recombinant fusion protein with multiple targets, increased affinity, and the capacity to prevent the growth of new blood vessels (15). Su et al. first evaluated the effect and safety of using Conbercept as an adjuvant to PPV in treating PDR, showing that intravitreal Conbercept (IVC) before PPV effectively accelerates visual recovery and reduces non-clearing vitreous hemorrhage (16). Subsequent meta-analyses also demonstrated the therapeutic efficacy and safety of IVC injection as an adjuvant to PPV in treating PDR.

Nevertheless, the intervals of IVC injection as an adjuvant to PPV varies in clinical practice, such as injection before PPV and immediate injection at the end of PPV. Currently, the impact of the intervals of IVC injection on intraoperative and postoperative outcomes in PDR patients undergoing PPV remains unknown because previous meta-analyses did not differentiate the intervals of IVC injection. Therefore, we conducted this network meta-analysis to evaluate the differences in therapeutic efficacy and safety between different intervals of IVC injection as an adjuvant to PPV in treating patients with PDR.

## Subjects and methods

### Study design

We conducted an NMA following the Preferred Reporting Items for Systematic Reviews and Meta-analyses guidelines (PRISMA) extension statement for reporting network meta-analysis (17). Since the statistical analysis was done using the published data, ethical approval and the patient’s informed consent were unnecessary. We have registered the present network meta-analysis in PROSPERO with registration number CRD42022361537.

### Eligibility criteria

The following criteria guided our selection of eligible studies: (a) adult patients received pars plana vitrectomy (PPV) for PDR; (b) PPV combined with intravitreal Conbercept (IVC) compared with PPV without Conbercept or each other; (c) the dose of IVC was limited to 0.5 mg, but there was no restriction on the type of PPV (23G, 25G, and 27G); (d) studies reported at least one of the best corrected visual acuity (BCVA), operation time, central macular thickness, intraoperative bleeding, iatrogenic retinal breaks, endodiathermy application, silicone oil tamponade, and postoperative vitreous hemorrhage (VH); and (e) only RCTs with full texts were considered.

Ineligible studies were excluded based on the following criteria: (a) papers reporting data from the same study, (b) studies without reporting IVC dose, (c) studies combining IVC with other drugs, (d) studies only report a broad time range to conduct IVC, (e) abstracts, letters to the editor, case reports, cell studies, animal studies, and reviews.

### Literature retrieval

Two independent authors searched the PubMed, EMBASE, and Cochrane library databases for relevant publications from their inception until August 11, 2022, using a combination of the terms “diabetic retinopathy” and “Conbercept.” We also manually searched relevant review articles and the reference lists of all eligible studies to identify additional studies. **Supplementary Table 1** shows the detailed search strategies for three targeted databases. Disagreements were resolved through discussions between the two authors until a consensus was reached.

### Study selection

In the following three steps, two authors independently selected eligible studies. First, we used the EndNote X9 software to remove duplicate studies. Second, we excluded irrelevant studies after reviewing the title and abstract. Third, ineligible studies were further identified by checking the full texts of the remaining studies. Disagreements between the two authors were resolved through discussions until an agreement was reached.

### Data extraction

Two authors extracted the following data independently: first author’s name, country, publication year, sample size, the proportion of males, patients’ mean age, duration of diabetes, details of comparisons and interventions, and follow-up duration. Only information from the final follow-up was extracted for the meta-analysis. We filled in missing data by contacting the corresponding author *via* email if necessary. The two authors debated any conflicts until they agreed.

### Outcomes of interest

The primary outcomes were the BCVA expressed as a logarithm of the minimal angle of resolution (*LogMAR*) at the final follow-up, the operation time, and the central macular thickness; however, intraoperative bleeding, iatrogenic retinal breaks, endodiathermy application, silicone oil tamponade, and postoperative vitreous hemorrhage were secondary outcomes.

### Assessment of risk of bias

Two independent authors used the revised Cochrane risk-of-bias tool for randomized trials (RoB2) to assess the risk of bias (18) from the randomization process, derivation for intended interventions, the missing outcome data, measurement of the outcome, selection of the reported results, and the overall results. Each domain was assigned a rating of low, no information, some concerns, or high risk. The two authors talked things out until they came to an agreement to resolve any disagreements.

### Data analysis

The estimates of continuous and binary variables were expressed using mean difference (MD) and risk ratio (RR) with corresponding 95% confidence intervals (CI), respectively. We first assessed transitivity between studies by determining whether there was an insignificant difference in major clinical and methodological characteristics between comparisons (19, 20). Then, we used the design-by-treatment interaction method (21) and the node-splitting method (22) to examine global and local consistency, respectively. Additionally, we also used the node-splitting method (23, 24) to examine the loop-closed inconsistency. We used random network meta-analysis to compare the efficacy of different regimens regardless of statistical heterogeneity (25). Furthermore, the relative rankings of all regimens were determined by estimating ranking probabilities using the surface under the cumulative ranking (SUCRA) (26). Finally, we created a comparison-adjusted funnel plot to investigate the possibility of publication bias (27). STATA 14.0 was used for statistical analysis (StataCorp LP, College Station, Texas, USA) (28). The graphical tools created by Chaimani et al. (29) were used to present all results graphically.

## Results

### Literature retrieval and selection

**Figure 1:**
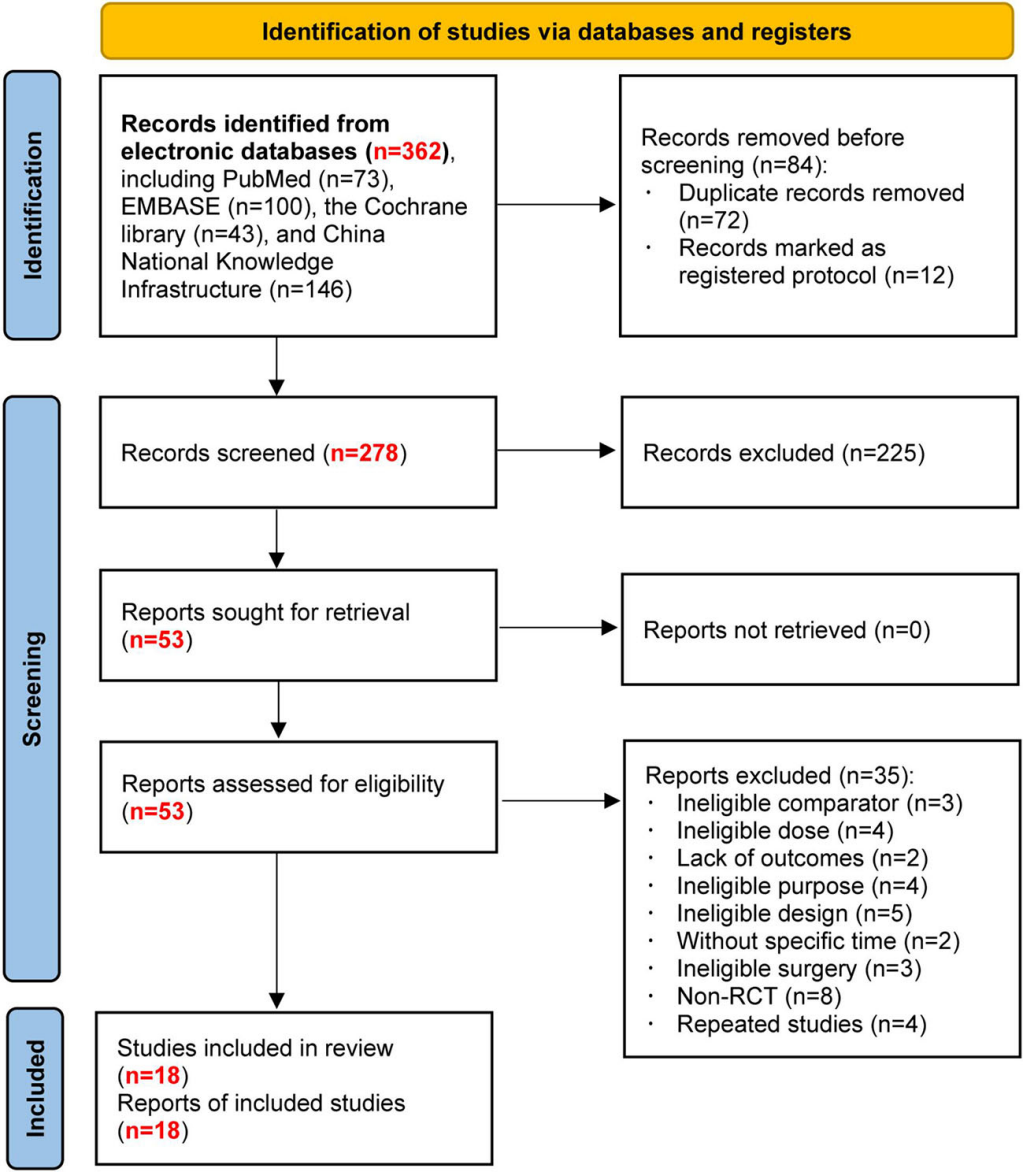
Flow diagram of study retrieval and selection.

**Figure 1** shows the flow diagram for retrieving and selecting studies. We identified 362 records from an electronic literature search and excluded 84 duplicates and registered protocols. After reviewing the abstracts, we eliminated 225 studies. Initially, 53 studies were potentially relevant. Among them, 35 studies were excluded after reading the full text. Finally, 18 studies were included in this network meta-analysis.

### Study characteristics

All studies (30–45) were performed in China and published between 2015 and 2021. The sample size of the individual study ranged from 31 to 111, with a total of 1149 patients. We defined it as short interval (SI) if the mean time of conducting IVC before PPV was ≤ 3 days, mid interval (MI) if the mean time of conducting IVC before PPV was > 3 days but ≤ 5 days, long interval (LI) if the mean time of conducting IVC before PPV was > 5 days but ≤ 7 days, and very long interval (VLI) if the mean time of conducting IVC before PPV was > 7 days but ≤ 9 days. Moreover, if IVC was conducted both before and at the end of PPV, we defined this strategy as perioperative IVC; however, the strategy was defined as intraoperative IVC if it was conducted immediately at the end of PPV. Overall, seven studies (32–34, 36, 40, 42, 46) compared LI with control, three studies (37, 38, 44) compared MI with control, three studies (35, 43, 47) compared SI with control, two studies (31, 39) compared intraoperative IVC with control, one study (41) compared VLI with SI, one study (45) compared MI with SI, and one study (30) compared MI with intraoperative and perioperative IVC. Detailed baseline characteristics of the included studies are presented in **Table 1**. We assessed transitivity based on publication year, sample size, male proportion, patients’ mean age, diabetes duration, and follow-up duration. The distribution of these six factors is insignificant across comparisons, as shown in **Supplemental Table 2**, demonstrating transitivity between comparisons.

**Table 1:** The baseline characteristics of eligible studies included in this network meta-analysis (n=18).

Study	Groups	Sample size, n	Males, n	Mean age, years	Duration of diabetes, years	Details of procedures	Follow-up duration
Li et al., 2020	Control	20	9	56.0±10.5	14.0±6.8	23G PPV without IVC	n.r.
	LI	20	13	51.1±11.6	10.9±7.7	0.5mg IVC at 7 days before 23G PPV	
Li et al., 2021	Control	38	16	51.9±9.2	10.2±3.3	27G PPV without IVC	6 months
	LI	39	20	52.1±8.5	9.9±2.7	0.5mg IVC at 6-7 days before 27G PPV	
Lin et al., 2018	Control	47	31	58.9±7.8	9.6±2.6	23G PPV without IVC	6 months
	LI	47	29	56.3±9.6	10.4±2.2	0.5mg IVC at 5-7 days before 23G PPV	
Luo et al., 2021	Control	42	22	62.5±3.7	5.0±1.5	23G PPV without IVC	1 month
	SI	42	21	62.1±3.5	6.0±1.6	0.5mg IVC at 3 days before 23G PPV	
Luo et al., 2018	Control	16	7	57.7±11.3	n.r.	23G PPV without IVC	3 months
	LI	15	5	57.0±13.1	n.r.	0.5mg IVC at 7 days before 23G PPV	
Ou et al., 2021	Control	37	14	57.4±11.0	8.89±2.23	23G PPV without IVC	3 months
	MI	38	15	56.7±12.7	8.71±1.98	0.5mg IVC at 5 days before 23G PPV	
Ran et al., 2016	Control	29	15	49.5±5.4	n.r.	23G PPV without IVC	n.r.
	MI	27	13	47.5±3.2	n.r.	0.5mg IVC at 5 days before 23G PPV	
Shang et al, 2018	Control	30	16	55.6±5.9	15.1±1.9	23G PPV without IVC	3 months
	LI	30	18	54.2±6.3	14.4±1.7	0.5mg IVC at 7 days before 23G PPV	
Su et al., 2016	Control	18	n.r.	n.r.	n.r.	23G PPV without IVC	1 month
	LI	18	n.r.	n.r.	n.r.	0.5mg IVC at 7 days before 23G PPV	
Sun et al., 2017	Control	42	23	45.2±8.9	10.03±5.74	25G PPV without IVC	3 months
	SI	41	22	48.7±9.5	9.86±6.07	0.5mg IVC at 3 days before 25G PPV	
Sun et al., 2015	Control	28	18	57.4±3.3	10.0±1.3	23G PPV without IVC	6 months
	MI	28	18	51.2±3.2	10.0±1.4	0.5mg IVC at 3-5 days before 23G PPV	
Yang et al., 2016	Control	53	24	49.6±8.7	15.9±4.8	23G PPV without IVC	3 months
	SI	54	27	48.6±8.2	16.7±4.5	0.5mg IVC at 3 days before 23G PPV	
Zhao et al., 2018	Control	18	22	46.9±12.3	n.r.	23G PPV without IVC	3 months
	LI	18				0.5mg IVC at 7 days before 23G PPV	
Jiang et al., 2020	Control	15	10	53.5±9.6	9.88±8.52	23G PPV without IVC	6 months
	Intraoperative	15	6	55.5±9.9	13.19±8.08	0.5mg IVC at the end of the 23G PPV	
Ren et al., 2019	Control	22	15	46-80	1204±6.05	25G PPV without IVC	6 months
	Intraoperative	23	16	28-69	10.36±4.17	0.5mg IVC at the end of the 25G PPV	
Gao et al., 2020	MI	34	14	50.8±13.5	14.5±5.2	0.5mg IVC at 3-5 days before 23G PPV	6 months
	Intraoperative	35	16	54.0±14.8	12.9±5.2	0.5mg IVC at the end of the 25G PPV	
	Perioperative	29	16	52.6±14.6	12.7±5.2	0.5mg IVC at 3-5 days before and after 23G PPV	
Shi et al., 2020	SI	26	13	52.7±9.0	8.9±5.9	0.5mg IVC at 2-3 days before 25G PPV	6 months
	VLI	21	9	52.1±10.5	10.3±5.9	0.5mg IVC at 7-8 days before 25G PPV	
Wen et al., 2019	Control	30	18	59.0±6.2	6.87±1.69	25G PPV without IVC	3 months
	SI	30	17	61.3±7.1	6.91±1.71	0.5mg IVC at 3 days before 25G PPV	
	MI	30	20	60.7±6.6	6.59±1.61	0.5mg IVC at 5 days before 25G PPV	

PPV, pars plana vitrectomy; IVC, intravitreal conbercept; VLI, very long interval; LI, long interval; MI, mid interval; SI, short interval; n.r., not reported.

### Risk of bias of eligible studies

Even though all eligible studies were RCTs, only seven studies (31, 32, 34, 36, 37, 39, 45) provided information on the randomization process. There was insufficient data to determine whether results are biased by deviations from the intended intervention and bias in outcome measures. Four studies were rated to be high risk due to incomplete outcome data. All studies were rated to be low risk in selective outcome reporting. **Supplementary Figure 1** shows the details of the risk of bias assessment.

### Meta-analysis of BCVA

**Figure 2:**
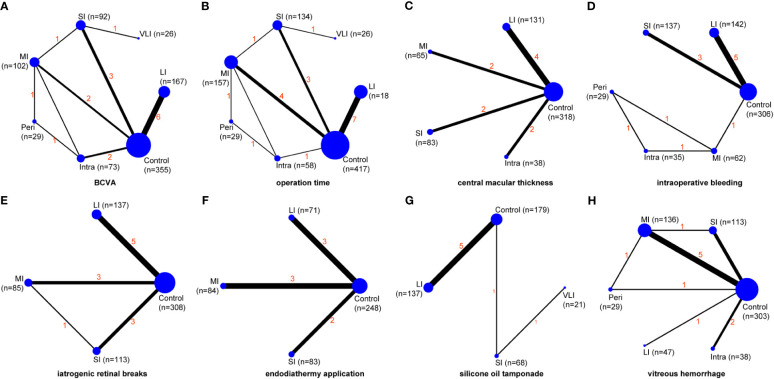
Network maps of evidence for all outcomes, including BCVA **(A)**, operation time **(B)**, central macular thickness **(C)**, intraoperative bleeding **(D)**, iatrogenic retinal breaks **(E)**, endodiathermy application **(F)**, silicone oil tamponade **(G)**, and vitreous hemorrhage **(H)**. VLI, very long interval; LI, long interval; MI, mid interval; SI, short interval; Intra, intraoperative; Peri, perioperative; BCVA, best corrected visual acuity.

**Figure 3:**
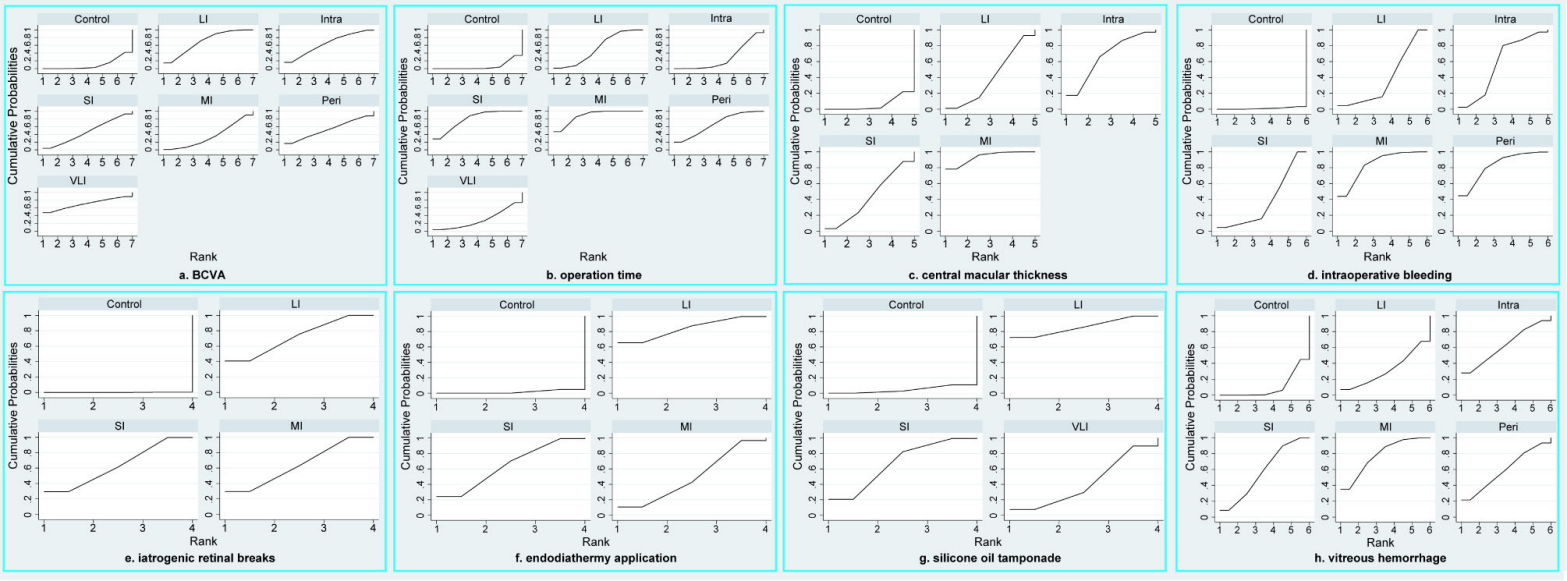
SUCRA plots of all outcomes, including BCVA **(A)**, operation time **(B)**, central macular thickness **(C)**, intraoperative bleeding **(D)**, iatrogenic retinal breaks **(E)**, endodiathermy application **(F)**, silicone oil tamponade **(G)**, and vitreous hemorrhage **(H)**. VLI, very long interval; LI, long interval; MI, mid interval; SI, short interval; Intra, intraoperative; Peri, perioperative; BCVA, best corrected visual acuity.

BCVA was reported in fourteen studies (30, 31, 33–37, 39–43, 45, 46), involving a total of 886 patients and seven regimens (**Figure 2A**). We used an inconsistency model to estimate the relative efficacy of various regimens because inconsistency examination revealed the existence of global inconsistency (**Supplementary Figure 2A**) and local inconsistency (**Supplementary Table 3**). As shown in **Table 2**, EP was associated with better BCVA compared to the control regimen (MD: -0.29, 95%CI: -0.44 to -0.15); however, no statistical difference was found in the remaining comparisons. Nevertheless, according to ranking probabilities based on SUCRA, VLI ranked first (70.6%), followed by LI (69.6%), intraoperative IVC (64.0%), perioperative IVC (53.0%), SI (47.3%), MI (35.4%), and control (10.2%), as shown in **Figure 3A**.

**Table 2:** Network meta-analysis of all regimens in terms of all outcomes.

Comparisons	BCVA, LogMAR	Operation time, min	Central macular thickness, μm	Intraoperation bleeding, n	Iatrogenic retinal breaks, n	Endodiathermy application, n	Silicone oil tamponade, n	Vitreous hemorrhage, n
VLI vs Control	-0.38 (-1.04, 0.28)	-1.56 (-29.21, 26.10)	n.a.	n.a.	n.a.	n.a.	**3.27 (1.84,5.83)**	n.a.
VLI vs LI	-0.09 (-0.76, 0.59)	16.91 (-11.91, 45.74)	n.a.	n.a.	n.a.	n.a.	2.33 (0.99,5.49)	n.a.
VLI vs MI	-0.25 (-0.96, 0.46)	26.10 (-2.50, 54.70)	n.a.	n.a.	n.a.	n.a.	n.a.	n.a.
VLI vs SI	-0.19 (-0.77, 0.39)	17.81 (-7.35, 42.97)	n.a.	n.a.	n.a.	n.a.	1.88 (0.70,5.10)	n.a.
VLI vs Intra	-0.11 (-0.81, 0.60)	6.92 (-24.37, 38.21)	n.a.	n.a.	n.a.	n.a.	n.a.	n.a.
VLI vs Peri	-0.16 (-0.95, 0.63)	26.10 (-2.50, 54.70)	n.a.	n.a.	n.a.	n.a.	n.a.	n.a.
Peri vs Control	-0.19 (-0.50, 0.12)	**-21.81 (-42.52, -1.10)**	n.a.	**0.04 (0.00, 0.76)**	n.a.	n.a.	n.a.	0.34 (0.07,1.55)
Peri vs LI	0.07 (0.38, 0.53)	-3.34 (-25.59, 18.92)	n.a.	0.16 (0.01, 3.07)	n.a.	n.a.	n.a.	0.51 (0.05,5.14)
Peri vs MI	-0.09 (-0.43, 0.25)	5.85 (-13.73, 25.43)	n.a.	1.01 (0.38, 2.65)	n.a.	n.a.	n.a.	1.43 (0.29,7.03)
Peri vs SI	-0.03 (-0.56, 0.50)	-2.44 (-25.51, 20.63)	n.a.	0.15 (0.01, 3.10)	n.a.	n.a.	n.a.	0.93 (0.17,4.94)
Peri vs Intra	0.05 (-0.45, 0.56)	-13.33 (-30.06, 6.41)	n.a.	0.60 (0.26, 1.41)	n.a.	n.a.	n.a.	1.09 (0.12,10.02)
MI vs Control	-0.09 (-0.50, 0.32)	**-27.66 (-37.19, -18.13)**	**-114.20 (-179.93, -48.48)**	**0.04 (0.00,0.65)**	**0.28 (0.12, 0.65)**	0.42 (0.17, 1.08)	n.a.	**0.24 (0.10,0.57)**
MI vs LI	0.16 (-0.14, 0.47)	-9.19 (-21.71, 3.34)	-80.65 (-161.67, 0.37)	0.16 (0.01,2.60)	1.12 (0.38, 3.28)	2.04 (0.42, 9.79)	n.a.	0.36 (0.05,2.51)
MI vs SI	0.06 (-0.35, 0.47)	-8.29 (-21.88, 5.30)	-77.54 (-170.22, 15.13)	0.15 (0.01, 2.63)	0.99 (0.31, 3.18)	1.30 (0.35, 4.82)	n.a.	0.65 (0.21,1.96)
MI vs Intra	0.14 (-0.23, 0.52)	**-19.18 (-34.50, -3.85)**	-45.31 (-143.42, 52.81)	0.60 (0.27,1.34)	n.a.	n.a.	n.a.	0.76 (0.12,4.80)
SI vs Control	-0.13 (-0.40, 0.14)	**-19.37 (-30.84, -7.89)**	-36.66 (-101.98, 28.66)	**0.27 (0.13,0.54)**	**0.29 (0.12, 0.68)**	**0.33 (0.13, 0.83)**	1.74 (0.55,5.49)	**0.37 (0.18,0.74)**
SI vs LI	0.10 (-0.24, 0.45)	-0.90 (-14.96, 13.17)	-3.11 (-83.76, 77.55)	1.03 (0.45,2.40)	1.14 (0.38, 3.42)	1.57 (0.33, 7.45)	1.24 (0.74,2.06)	0.55 (0.08,3.59)
SI vs Intra	0.08 (-0.32, 0.49)	-10.89 (-29.49, 7.71)	32.23 (-65.36, 129.83)	4.00 (0.20,78.47)	n.a.	n.a.	n.a.	1.17 (0.20,6.85)
Intra vs LI	0.02 (-0.28, 0.32)	9.99 (-7.29, 27.28)	-35.34 (-121.83, 51.15)	0.26 (0.01,4.84)	n.a.	n.a.	n.a.	0.47 (0.04,5.06)
Intra vs Control	0.19 (-0.18, 0.56)	-8.48 (-23.73, 6.77)	-68.89 (-141.48, 3.69)	0.07 (0.00,1.20)	n.a.	n.a.	n.a.	0.31 (0.06,1.58)
LI vs Control	**-0.29 (-0.44, -0.15)**	**-18.47 (-26.61, -10.34)**	-33.55 (-80.87, 13.77)	**0.26 (0.16,0.41)**	**0.25 (0.13, 0.50)**	**0.21 (0.06, 0.73)**	1.40 (0.50,3.94)	0.67 (0.12,3.81)

BCVA, best corrected visual acuity; LogMAR, logarithm of the minimal angle of resolution; VLI, very long interval; LI, long interval; MI, mid interval; SI, short interval; Intra, intraoperative; Peri, perioperative.

Numbers in bold font indicates statistical significance. n.a., not applicable.

### Meta-analysis of operation time

Operation time was reported in sixteen studies (30, 32–46), involving a total of 1008 patients and seven regimens (**Figure 2B**). We used a consistency model to estimate the relative efficacy of various regimens because inconsistency examination revealed the absence of global inconsistency (**Supplementary Figure 2B**) and local inconsistency (**Supplementary Table 3**). All regimens, except for VLI and intraoperative IVC, were associated with fewer operation times when compared to the control regimen, as shown in **Table 2**; however, we found no statistical difference in the remaining comparisons. Meanwhile, MI outperformed intraoperative IVC in reducing operation time (MD: -19.74, 95%CI: -33.31 to -6.17). According to ranking probabilities based on SUCRA, MI ranked first (88.2%), followed by SI (79.3%), perioperative IVC (67.2%), LI (52.3%), VLI (29.3%), intraoperative IVC (27.4%), and control (6.3%), as shown in **Figure 3B**.

### Meta-analysis of central macular thickness

Central macular thickness was reported in ten studies (31, 33–40, 43), involving 635 patients and five regimens (**Figure 2C**). Because inconsistency examination does not apply to this outcome, we used a consistency model to estimate the relative efficacy of various regimens. As shown in **Table 2**, MI was better than the control regimen in improving central macular thickness (MD: -114.20, 95%CI: -179.93 to -48.48); however, no statistical difference was found in the remaining comparisons. According to ranking probabilities based on SUCRA, MI ranked first (93.4%), followed by intraoperative IVC (66.8%), SI (43.2%), LI (40.7%), and control (5.9%), as shown in **Figure 3C**.

### Meta-analysis of intraoperative bleeding

Intraoperative bleeding was reported in ten studies (31, 36–41, 45), involving 711 patients and six regimens (**Figure 2D**). Global inconsistency examination is not applicable to this outcome, but the assumption of the presence of local inconsistency was rejected (**Supplementary Table 3**). Therefore, we used a consistency model to estimate the relative efficacy of various regimens. As shown in **Table 2**, except for intraoperative IVC, all regimens were associated with fewer intraoperative bleeding when compared to control regimen; however, no statistical difference was found in the remaining comparisons. According to ranking probabilities based on SUCRA, MI ranked first (84.2%), followed by perioperative IVC (82.7%), intraoperative IVC (57.0%), LI (38.1%), SI (37.0%), and control (1.1%), as shown in **Figure 3D**.

### Meta-analysis of iatrogenic retinal breaks

The data of iatrogenic retinal breaks were reported in ten studies (33–36, 38, 42–46), involving 643 patients and four regimens (**Figure 2E**). We used a consistency model to estimate the relative efficacy of various regimens because inconsistency examination revealed the absence of global inconsistency (**Supplementary Figure 2C**) and local inconsistency (**Supplementary Table 3**). All regimens were associated with fewer iatrogenic retinal breaks when compared to the control regimen, as shown in **Table 2**; however, no statistical difference was found in the remaining comparisons. According to ranking probabilities based on SUCRA, LI ranked first (72.1%), followed by MI (64.2%), SI (63.5%), and control (0.1%), as shown in **Figure 3E**.

### Meta-analysis of endodiathermy application

Endodiathermy application was reported in eight studies (32, 34, 35, 37, 38, 42, 43, 46), involving a total of 486 patients and four regimens (**Figure 2F**). Because inconsistency examination is not applicable to this outcome, we used a consistency model to estimate the relative efficacy of various regimens. As shown in **Table 2**, except for MI, SI and LI were associated with fewer endodiathermy applications compared to the control regimen; however, no statistical difference was found in the remaining comparisons. According to ranking probabilities based on SUCRA, LI ranked first (84.0%), followed by SI (64.5%), MI (49.9%), and control (1.6%), as shown in **Figure 3F**.

### Meta-analysis of silicone oil tamponade

Silicone oil tamponade was reported in seven studies (33–36, 41, 42, 46), involving 405 patients and four regimens (**Figure 2G**). Because inconsistency examination is not applicable to this outcome, we used a consistency model to estimate the relative efficacy of various regimens. As shown in **Table 2**, VLI was associated with more silicone oil tamponade compared to the control regimen (RR: 3.27, 95%CI, 1.84 to 5.83); however, no statistical difference was found in the remaining comparisons. According to ranking probabilities based on SUCRA, LI ranked first (86.0%), followed by SI (67.4%), VLI (42.1%), and control (4.5%), as shown in **Figure 3G**.

### Meta-analysis of postoperative vitreous hemorrhage

Postoperative vitreous hemorrhage was reported in ten studies (30, 31, 34, 35, 37–39, 43–45), involving a total of 666 patients and six regimens (**Figure 2H**). We used a consistency model to estimate the relative efficacy of various regimens because inconsistency examination revealed the absence of global inconsistency (**Supplementary Figure 2D**) and local inconsistency (**Supplementary Table 3**). MI and SI were associated with fewer postoperative vitreous hemorrhages compared to the control regimen, as shown in **Table 2**; however, no statistical difference was found in the remaining comparisons. According to ranking probabilities based on SUCRA, MI ranked first (77.9%), followed by intraoperative IVC (62.7%), perioperative IVC (59.4%), SI (57.6%), LI (32.0%), and control (10.3%), as shown in **Figure 3H**.

### Loop-closed inconsistency

Among eight target outcomes, the evidence maps of four outcomes covered loop-closed. As shown in **Supplementary Table 4**, loop-closed inconsistency was present for BCVA but not for operation time, iatrogenic retinal breaks, and postoperative vitreous hemorrhage.

### Publication bias

As shown in **Supplementary Figure 3**, all results reject the hypothesis of the existence of a small study effect, as all plots were visually symmetric.

## Discussion

As far as we know, this is the first study to ascertain the comparative efficacy of different timings of conducting IVC as an adjuvant to PPV on PDR by introducing the network meta-analysis technique. In the present network meta-analysis, the pooled results showed that the application of IVC in patients with PDR immediately after the PPV does not achieve additional therapeutic benefit. However, the application of IVC in patients with PDR before PPV, especially long and mid intervals, significantly increases the efficacy and safety of PPV.

The current network meta-analysis suggests that clinical practitioners may consider administering Conbercept before PPV to improve intraoperative and postoperative outcomes in patients with PDR. However, overly long interval between administration of IVC and PPV is associated with an increased need for silicone oil tamponade, so clinical workers need to weigh carefully regarding the timing of preoperative application. There is no consensus about the exact reasons for the different treatment responses at different intervals of IVC administration before PPV. Previous studies reveal possible effects of various intervals of performing IVC on clinical outcomes in patients undergoing PPV. Du and colleagues observed an immediate and rapid increase in the concentration when injecting Conbercept to treat hyperglycemic mouse eyes, with a decrease beginning on the seventh day after injection (48). Previous studies also suggested that administration of IVC seven days before PPV benefits achieving the best surgical outcomes (49, 50). This evidence partially explains why very-early preoperative IVC did not produce better surgical outcomes than other strategies. However, the specific reasons for the differences between preoperative, intraoperative, and perioperative IVC remain inconclusive. As a result, future research should investigate the underlying mechanisms by which IVC administration results in different clinical outcomes in PDR patients receiving PPV at different intervals.

To date, three meta-analyses (51–53) have investigated IVC’s therapeutic efficacy and safety on PPV for patients with PDR. The meta-analysis by Pranata and Vania (52) concluded that, compared to PPV alone, PPV combined with IVC was associated with greater improvement in BCVA, better intraoperative outcome, and less postoperative vitreous hemorrhage. The efficacy and safety of preoperative IVC as an adjunct to PPV in the treatment of PDR were also assessed in a meta-analysis by Si et al. (53). The results of a combined study of 23 studies (including 11 RCTs, 2 cohort studies, and 10 case-control studies) showed that preoperative IVC significantly shortened the average operation time and decreased the incidences of intraoperative bleeding, iatrogenic retinal breaks, and postoperative vitreous hemorrhage. We should note that two previous meta-analyses integrated the results from studies with different types of designs to estimate the efficacy and safety of IVC, which inevitably introduced bias to pooled results. Following the previous meta-analyses, another meta-analysis (51) with RCTs was performed to evaluate IVC’s efficacy in PPV for patients with PDR. The pooled results of eight studies suggested that IVC was associated with less intraoperative bleeding and endodiathermy applications, shorter surgical time, and better BCVA outcomes. Overall, three previous meta-analyses consistently supported IVC’s therapeutic efficacy and safety in PPV for patients with PDR. Although the previous meta-analysis noted the preoperative and intraoperative application of IVC, separate analyses were not performed according to the intervals of administering IVC. More importantly, the differences between preoperative and intraoperative IVC were not evaluated.

In contrast to earlier network meta-analyses, the current one only used RCTs to evaluate therapeutic efficacy and safety, greatly reducing the bias introduced by the study design. Furthermore, our network meta-analysis first designed a separate analysis to investigate the role of different intervals of administering IVC in the treatment of PDR and found that intraoperative application of IVC did not add additional benefits to PPV in treating patients with PDR. Finally, the present network meta-analysis also first classified the preoperative application of IVC into four sub-phases, including very long interval, long interval, mid interval, and short interval. It is noted that very long interval was found to have no additional therapeutic benefits to PPV but was associated with more application of silicone oil tamponade.

In addition, compared to previous meta-analyses, the current network meta-analysis also offers the following advantages in terms of methodology: (a) We retrieved all currently available eligible studies by employing a thorough literature retrieval strategy; (b) We could estimate the relative differences of various intervals of preoperative IVC using network meta-analysis; and (c) All the regimens were ranked using the SUCRA method, making it easier to choose the best regimen for clinical use.

Our pooled results should be interpreted with caution due to the following limitations: (a) This network meta-analysis only included a small number of eligible studies with small sample sizes, which may have a significant negative impact on the robustness of all pooled results; (b) Because all studies were conducted in China, results should be cautiously used in other clinical contexts before being further validated; (c) There was inconsistency between direct and indirect evidences which were used to estimate the relative efficacy in improving BCVA of all regimens; therefore, the pooled result of this outcome should be cautiously interpreted; (d) Three types of PPV were used in eligible studies; however, we could not perform subgroup analysis to eliminate the negative impact of type of PPV on the pooled results because there were limited studies were included in this network meta-analysis; and (e) Only two of the eight outcomes we examined covered all regimens, and it worth noting that there was no statistical difference between mid interval and perioperative IVC in terms of postoperative vitreous hemorrhage although ranking probability suggested more higher ranking for mid interval. SUCRA cannot show whether the difference between treatments is clinically meaningful. Therefore, more studies with larger sample sizes are required to evaluate the difference between different strategies, especially in postoperative vitreous hemorrhage between mid interval and perioperative IVC.

Our findings showed that using intraoperative IVC as an adjuvant to PPV does not achieve additional benefits for treating PDR. But preoperative IVC as an adjuvant to PPV, especially long and mid intervals of injecting IVC before performing PPV, achieves significant intraoperative and postoperative benefits in treating PDR, except for very long interval. However, additional studies are undoubtedly needed to validate our findings further.

## Author contributions

WW and CQ carried out the studies, participated in collecting data, and drafted the manuscript. CQ and HY performed the statistical analysis and participated in its design. WW and CQ participated in acquisition, analysis, or interpretation of data and draft the manuscript. All authors contributed to the article and approved the submitted version.

## Supplementary material

Supplementary Figure 1 Risk of bias assessment based on traffic light plot **(A)** and summary plot **(B)**.

Supplementary Figure 2 Global consistency model test for BCVA **(A)**, operation time **(B)**, iatrogenic retinal breaks **(C)**, and vitreous hemorrhage **(D)**. BCVA; best corrected visual acuity.

Supplementary Figure 3 Comparison-adjusted funnel plots of all outcomes.
