# Supplementary figures and images for: Network meta-analysis of intravitreal conbercept as an adjuvant to vitrectomy for proliferative diabetic retinopathy
Source: Front Endocrinol (Lausanne). 2023 Feb 22;14:1098165. doi: 10.3389/fendo.2023.1098165 (PMC9989469; doi:10.3389/fendo.2023.1098165)

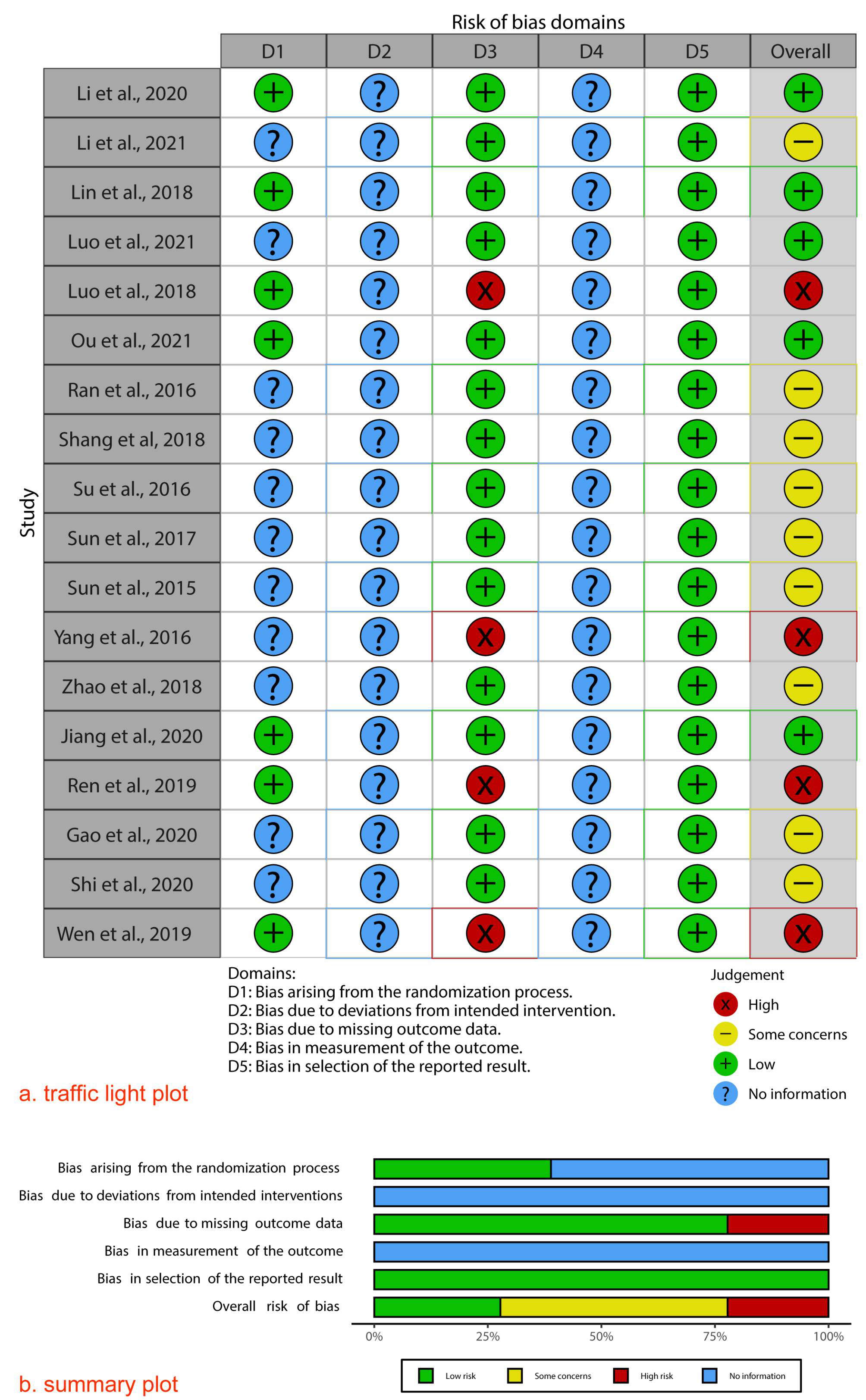

Supplement: Supplementary Figure 1 — Risk of bias assessment based on traffic light plot (A) and summary plot (B). [file Image_1.tif]

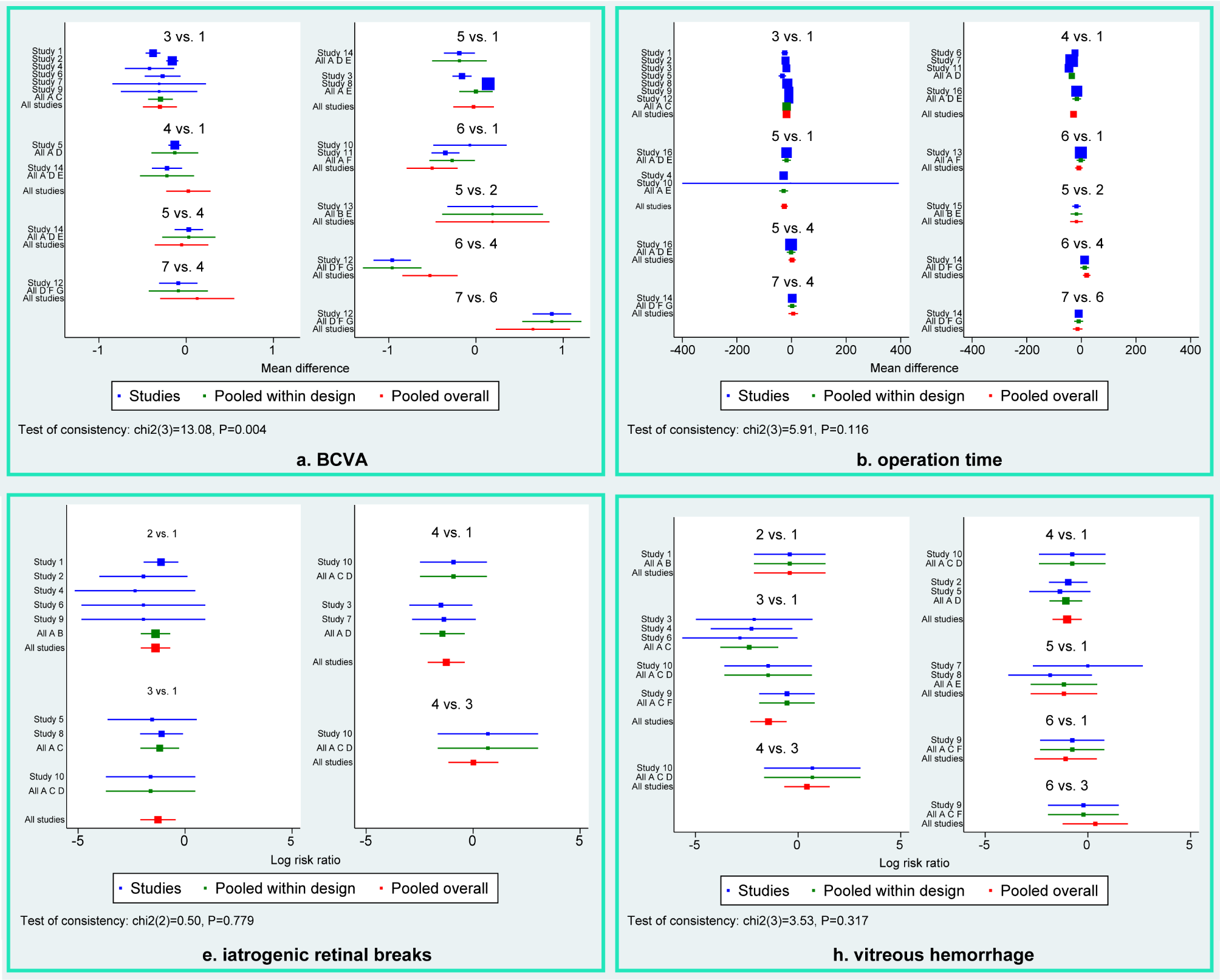

Supplement: Supplementary Figure 2 — Global consistency model test for BCVA (A), operation time (B), iatrogenic retinal breaks (C), and vitreous hemorrhage (D). BCVA; best corrected visual acuity. [file Image_2.tif]

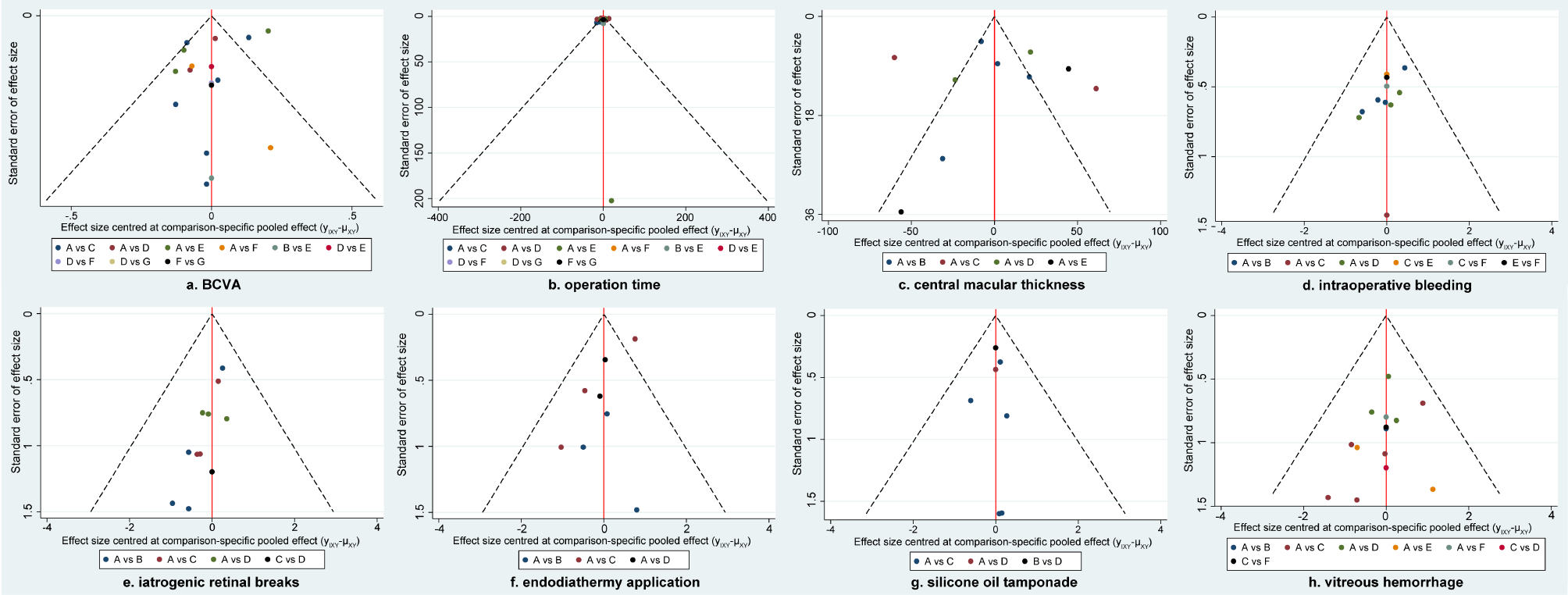

Supplement: Supplementary Figure 3 — Comparison-adjusted funnel plots of all outcomes. [file Image_3.tif]
