# Supplementary material for: Network meta-analysis of intravitreal conbercept as an adjuvant to vitrectomy for proliferative diabetic retinopathy
Source: Front Endocrinol (Lausanne). 2023 Feb 22;14:1098165. doi: 10.3389/fendo.2023.1098165 (PMC9989469; doi:10.3389/fendo.2023.1098165)
Supplement: Supplementary file 4 [file Table_1.docx]

**Supplementary Table 1.** Detailed search strategies of target English databases.

1. *Search strategy of PubMed*

| No. | Search Details | Results |
| --- | --- | --- |
| 7 | ("Diabetic Retinopathy"[MeSH Terms] OR ("Diabetic Retinopathy"[Title/Abstract] OR "diabetic retinopathies"[Title/Abstract])) AND ("KH902 fusion protein"[Supplementary Concept] OR ("conbercept"[Title/Abstract] OR "KH902"[Title/Abstract])) | 73 |
| 6 | "KH902 fusion protein"[Supplementary Concept] OR "conbercept"[Title/Abstract] OR "KH902"[Title/Abstract] | 265 |
| 5 | "conbercept"[Title/Abstract] OR "KH902"[Title/Abstract] | 259 |
| 4 | "KH902 fusion protein"[Supplementary Concept] | 139 |
| 3 | "Diabetic Retinopathy"[MeSH Terms] OR "Diabetic Retinopathy"[Title/Abstract] OR "diabetic retinopathies"[Title/Abstract] | 38,190 |
| 2 | "diabetic retinopathy"[Title/Abstract] OR "diabetic retinopathies"[Title/Abstract] | 27,641 |
| 1 | "Diabetic Retinopathy"[MeSH Terms] | 28,200 |

1. *Search strategy of EMBASE*

| No. | Query | Results |
| --- | --- | --- |
| #7 | #3 AND #6 | 100 |
| #6 | #4 OR #5 | 446 |
| #5 | 'conbercept'/exp | 431 |
| #4 | conbercep:ti,ab,kw OR kh902:ti,ab,kw | 30 |
| #3 | #1 OR #2 | 58770 |
| #2 | 'diabetic retinopathy'/exp | 51746 |
| #1 | 'diabetic retinopathy':ti,ab,kw OR 'diabetic retinopathies':ti,ab,kw | 39496 |

1. *Search strategy of CENTRAL*

| No. | Search | Hits |
| --- | --- | --- |
| #1 | (diabetic retinopathy):ti,ab,kw OR (diabetic retinopathies):ti,ab,kw | 4491 |
| #2 | MeSH descriptor: [Diabetic Retinopathy] explode all trees | 1575 |
| #3 | #1 or #2 | 4491 |
| #4 | (conbercept):ti,ab,kw OR (KH902):ti,ab,kw | 119 |
| #5 | #3 and #4 | 43 |
