# Supplementary material for: Network meta-analysis of intravitreal conbercept as an adjuvant to vitrectomy for proliferative diabetic retinopathy
Source: Front Endocrinol (Lausanne). 2023 Feb 22;14:1098165. doi: 10.3389/fendo.2023.1098165 (PMC9989469; doi:10.3389/fendo.2023.1098165)
Supplement: Supplementary file 5 [file Table_2.docx]

**Supplementary Table 2.** Transitivity assessment between studies included in this network meta-analysis

| Variables | Comparisons | Mean/Z | SE | p-value |
| --- | --- | --- | --- | --- |
| Publication year | Control vs VLI | -1.733 | 2.083 | 0.419 |
|  | Control vs LI | -0.162 | 0.872 | 0.855 |
|  | Control vs MI | 0.067 | 1114 | 0.953 |
|  | Control vs SI | -0.333 | 1.048 | 0.754 |
|  | Control vs Intra | -1.400 | 1.200 | 0.260 |
|  | Control vs Peri | -1.733 | 2.083 | 0.419 |
|  | VLI vs LI | 1.571 | 1.730 | 0.399 |
|  | VLI vs MI | 1.800 | 2.835 | 0.560 |
|  | VLI vs SI | 1.400 | 2.272 | 0.571 |
|  | VLI vs Intra | 0.333 | 0.667 | 0.667 |
|  | VLI vs Peri | 0.000 | n.a. | n.a. |
|  | LI vs MI | 0.229 | 1.207 | 0.854 |
|  | LI vs SI | -0.171 | 1.062 | 0.875 |
|  | LI vs Intra | -1.238 | 0.987 | 0.245 |
|  | LI vs Peri | -1.571 | 1.730 | 0.399 |
|  | MI vs SI | -0.400 | 1.483 | 0.794 |
|  | MI vs Intra | -1.467 | 1.563 | 0.384 |
|  | MI vs Peri | -1.800 | 2.835 | 0.560 |
|  | SI vs Intra | -1.067 | 1.260 | 0.430 |
|  | SI vs Peri | -1.400 | 2.272 | 0.571 |
|  | Intra vs Peri | -0.333 | 0.667 | 0.667 |
| Sample size | Control vs VLI | 9.313 | 12.183 | 0.456 |
|  | Control vs LI | 3.598 | 5.419 | 0.514 |
|  | Control vs MI | -1.087 | 5.486 | 0.845 |
|  | Control vs SI | -10.438 | 6.575 | 0.130 |
|  | Control vs Intra | 5.979 | 7.315 | 0.425 |
|  | Control vs Peri | 1.313 | 12.183 | 0.916 |
|  | VLI vs LI | -5.714 | 13.147 | 0.679 |
|  | VLI vs MI | -10.400 | 4.996 | 0.106 |
|  | VLI vs SI | -17.600 | 12.090 | 0.219 |
|  | VLI vs Intra | -3.333 | 11.624 | 0.801 |
|  | VLI vs Peri | -8.000 | n.a. | n.a. |
|  | LI vs MI | -4.686 | 5.828 | 0.440 |
|  | LI vs SI | -11.886 | 6.915 | 0.116 |
|  | LI vs Intra | 2.381 | 8.129 | 0.777 |
|  | LI vs Peri | -2.286 | 13.147 | 0.868 |
|  | MI vs SI | -7.200 | 5.340 | 0.215 |
|  | MI vs Intra | 7.067 | 5.041 | 0.211 |
|  | MI vs Peri | 2.400 | 4.996 | 0.656 |
|  | SI vs Intra | 14.267 | 7.831 | 0.118 |
|  | SI vs Peri | 9.600 | 12.090 | 0.472 |
|  | Intra vs Peri | -4.667 | 11.624 | 0.727 |
| Male proportion | Control vs VLI | 8.000 | 6.671 | 2.52 |
|  | Control vs LI | 0.000 | 3.693 | 1.000 |
|  | Control vs MI | 1.000 | 3.027 | 0.745 |
|  | Control vs SI | -3.000 | 3.226 | 0.365 |
|  | Control vs Intra | 4.333 | 4.046 | 0.301 |
|  | Control vs Peri | 1.000 | 6.671 | 0.883 |
|  | VLI vs LI | -8.000 | 9.706 | 0.456 |
|  | VLI vs MI | -7.000 | 3.194 | 0.094 |
|  | VLI vs SI | -11.000 | 5.797 | 0.131 |
|  | VLI vs Intra | -3.667 | 6.667 | 0.638 |
|  | VLI vs Peri | -7.000 | n.a. | n.a. |
|  | LI vs MI | 1.000 | 4.171 | 0.817 |
|  | LI vs SI | -3.000 | 4.615 | 0.534 |
|  | LI vs Intra | 4.333 | 5.817 | 0.484 |
|  | LI vs Peri | 1.000 | 9.706 | 0.923 |
|  | MI vs SI | -4.000 | 2.702 | 0.177 |
|  | MI vs Intra | 3.333 | 2.991 | 0.308 |
|  | MI vs Peri | 0.000 | 3.194 | 1.000 |
|  | SI vs Intra | 7.333 | 3.985 | 0.115 |
|  | SI vs Peri | 4.000 | 5.797 | 0.528 |
|  | Intra vs Peri | -3.333 | 6.667 | 0.667 |
| Mean age | Control vs VLI | 2.264 | 5.254 | 0.674 |
|  | Control vs LI | 1.431 | 2.314 | 0.544 |
|  | Control vs MI | 0.984 | 2.667 | 0.717 |
|  | Control vs SI | -0.316 | 2.855 | 0.913 |
|  | Control vs Intra | -0.386 | 3.703 | 0.919 |
|  | Control vs Peri | 1.764 | 5.254 | 0.742 |
|  | VLI vs LI | -0.833 | 4.040 | 0.845 |
|  | VLI vs MI | -1.280 | 5.760 | 0.835 |
|  | VLI vs SI | -2.580 | 7.257 | 0.740 |
|  | VLI vs Intra | -2.650 | 1.299 | 0.290 |
|  | VLI vs Peri | -0.500 | n.a. | n.a. |
|  | LI vs MI | -0.447 | 2.711 | 0.873 |
|  | LI vs SI | -1.747 | 3.162 | 0.594 |
|  | LI vs Intra | -1.817 | 2.810 | 0.542 |
|  | LI vs Peri | 0.333 | 4.040 | 0.937 |
|  | MI vs SI | -1.300 | 3.782 | 0.740 |
|  | MI vs Intra | -1.370 | 3.955 | 0.743 |
|  | MI vs Peri | 0.780 | 5.760 | 0.899 |
|  | SI vs Intra | -0.070 | 4.973 | 0.989 |
|  | SI vs Peri | 2.080 | 7.257 | 0.789 |
|  | Intra vs Peri | 2.150 | 1.299 | 0.346 |
| Duration of DM | Control vs VLI | 0.326 | 3.329 | 0.924 |
|  | Control vs LI | -0.774 | 1.725 | 0.661 |
|  | Control vs MI | 0.676 | 1.864 | 0.722 |
|  | Control vs SI | 0.9512 | 1.863 | 0.617 |
|  | Control vs Intra | -1.524 | 1.940 | 0.446 |
|  | Control vs Peri | -2.074 | 3.329 | 0.546 |
|  | VLI vs LI | -1.100 | 2.282 | 0.663 |
|  | VLI vs MI | 0.350 | 3.738 | 0.931 |
|  | VLI vs SI | 0.626 | 4.620 | 0.899 |
|  | VLI vs Intra | -1.850 | 1.798 | 0.412 |
|  | VLI vs Peri | -2.400 | n.a. | n.a. |
|  | LI vs MI | 1.450 | 1.959 | 0.487 |
|  | LI vs SI | 1.726 | 2.319 | 0.481 |
|  | LI vs Intra | -0.750 | 1.423 | 0.621 |
|  | LI vs Peri | -1.300 | 2.282 | 0.609 |
|  | MI vs SI | 0.276 | 2.594 | 0.918 |
|  | MI vs Intra | -2.200 | 2.116 | 0.346 |
|  | MI vs Peri | -2.750 | 3.738 | 0.515 |
|  | SI vs Intra | -2.476 | 2.599 | 0.378 |
|  | SI vs Peri | -3.026 | 4.620 | 0.548 |
|  | Intra vs Peri | -0.550 | 1.798 | 0.789 |
| Follow-up duration | Control vs VLI | -2.214 | 1.912 | 0.268 |
|  | Control vs LI | 0.119 | 0.918 | 0.898 |
|  | Control vs MI | -0.714 | 1.035 | 0.500 |
|  | Control vs SI | 0.586 | 0.955 | 0.548 |
|  | Control vs Intra | -2.214 | 1.094 | 0.061 |
|  | Control vs Peri | -2.214 | 1.912 | 0.268 |
|  | VLI vs LI | 2.333 | 2.124 | 0.322 |
|  | VLI vs MI | 1.500 | 1.936 | 0.495 |
|  | VLI vs SI | 2.800 | 1.960 | 0.226 |
|  | VLI vs Intra | **0.000** | **0.000** | **n.a.** |
|  | VLI vs Peri | 0.000 | n.a. | n.a. |
|  | LI vs MI | -0.833 | 1.215 | 0.512 |
|  | LI vs SI | 0.467 | 1.144 | 0.693 |
|  | LI vs Intra | -2.333 | 1.175 | 0.087 |
|  | LI vs Peri | -2.333 | 2.124 | 0.322 |
|  | MI vs SI | 1.300 | 1.184 | 0.308 |
|  | MI vs Intra | -1.500 | 1.025 | 0.203 |
|  | MI vs Peri | -1.500 | 1.936 | 0.495 |
|  | SI vs Intra | -2.800 | 1.067 | 0.192 |
|  | SI vs Peri | -2.800 | 1.960 | 0.226 |
|  | Intra vs Peri | 0.000 | 0.000 | n.a. |

DM, diabetes mellitus; VLI, very long interval; LI, long interval; MI, mid interval; SI, short interval; Intra, intraoperative; Peri, peri-operative; SE, standard error.
