# Supplementary material for: Network meta-analysis of intravitreal conbercept as an adjuvant to vitrectomy for proliferative diabetic retinopathy
Source: Front Endocrinol (Lausanne). 2023 Feb 22;14:1098165. doi: 10.3389/fendo.2023.1098165 (PMC9989469; doi:10.3389/fendo.2023.1098165)
Supplement: Supplementary file 6 [file Table_3.docx]

**Supplementary Table 3**. Local inconsistency assessment.

| Side | Direct | | Indirect | | Difference | | P | tau |
| --- | --- | --- | --- | --- | --- | --- | --- | --- |
|  | Coefficient | SE | Coefficient | SE | Coefficient | SE |  |  |
| BCVA | | | | | | | | |
| A C | n.a. | n.a. | n.a. | n.a. | n.a. | n.a. | n.a. | n.a. |
| A D | -0.170 | 0.100 | 0.529 | 0.178 | -0.699 | 0.206 | 0.001 | 0.126 |
| A E | -0.062 | 0.124 | 0.554 | 0.516 | -0.616 | 0.532 | 0.247 | 0.206 |
| A F | -0.277 | 0.127 | -1.098 | 0.189 | 0.821 | 0.228 | 0 | 0.122 |
| B E | 0.190 | 0.333 | -0.075 | 57.748 | 0.265 | 57.748 | 0.996 | 0.203 |
| D E | 0.031 | 0.233 | -0.146 | 0.239 | 0.177 | 0.334 | 0.596 | 0.218 |
| D F | -0.960 | 0.164 | -0.139 | 0.158 | -0.821 | 0.228 | 0 | 0.122 |
| D G | -0.090 | 0.166 | 1.553 | 0.426 | -1.643 | 0.456 | 0 | 0.122 |
| F G | 0.870 | 0.166 | -0.773 | 0.4266 | 1.643 | 0.456 | 0 | 0.122 |
| Operation time | | | | | | | | |
| A C | n.a. | n.a. | n.a. | n.a. | n.a. | n.a. | n.a. | n.a. |
| A D | -30.174 | 4.738 | -19.340 | 11.729 | -10.834 | 12.656 | 0.392 | 8.977 |
| A E | -23.468 | 6.340 | -48.666 | 17.985 | 25.198 | 19.094 | 0.187 | 8.462 |
| A F | -1.880 | 8.915 | -18.177 | 10.242 | 16.297 | 13.578 | 0.230 | 8.630 |
| B E | -17.810 | 11.868 | -50.660 | 1741.007 | 32.850 | 1741.040 | 0.985 | 8.850 |
| D E | -1.664 | 9.414 | 7.368 | 10.468 | -9.032 | 14.072 | 0.521 | 9.139 |
| D F | 12.250 | 9.206 | 28.559 | 9.982 | -16.309 | 13.579 | 0.230 | 8.630 |
| D G | 2.470 | 9.178 | 35.094 | 25.644 | -32.624 | 27.151 | 0.230 | 8.630 |
| F G | -9.780 | 9.331 | -42.374 | 25.483 | 32.5943 | 27.157 | 0.230 | 8.630 |
| Intraoperative bleeding | | | | | | | | |
| A B | n.a. | n.a. | n.a. | n.a. | n.a. | n.a. | n.a. | n.a. |
| A C | -3.219 | 1.418 | -0.214 | 54.037 | -3.004 | 54.055 | 0.956 | 0.000 |
| A D | n.a. | n.a. | n.a. | n.a. | n.a. | n.a. | n.a. | n.a. |
| C E | 0.510 | 0.410 | 6.519 | 108.049 | -6.009 | 108.050 | 0.956 | 0.000 |
| C F | 0.005 | 0.500 | 6.014 | 107.946 | -6.009 | 107.947 | 0.956 | 0.000 |
| E F | n.a. | n.a. | n.a. | n.a. | n.a. | n.a. | n.a. | n.a. |
| Iatrogenic retinal breaks | | | | | | | | |
| A B | n.a. | n.a. | n.a. | n.a. | n.a. | n.a. | n.a. | n.a. |
| A C | -1.249 | 0.423 | -2.3905 | 1.914 | 1.141 | 1.895 | 0.547 | 0.000 |
| A D | -1.273 | 0.443 | -0.229 | 2.2926 | -1.044 | 2.294 | 0.649 | 0.000 |
| C D | 0.710 | 1.156 | -0.250 | 0.704 | 0.960 | 1.364 | 0.481 | 0.000 |
| Vitreous hemorrhage | | | | | | | | |
| A B | n.a. | n.a. | n.a. | n.a. | n.a. | n.a. | n.a. | n.a. |
| A C | -1.427 | 0.452 | -2.055 | 1.819 | 0.629 | 1.803 | 0.727 | 0.000 |
| A D | -1.011 | 0.364 | -0.785 | 2.337 | -0.226 | 2.345 | 0.923 | 0.000 |
| A E | n.a. | n.a. | n.a. | n.a. | n.a. | n.a. | n.a. | n.a. |
| A F | -0.757 | 0.798 | -3.903 | 1.809 | 3.145 | 1.822 | 0.084 | 0.000 |
| C D | 0.744 | 1.158 | 0.341 | 0.640 | 0.403 | 1.311 | 0.759 | 0.000 |
| C F | -0.217 | 0.878 | 2.928 | 1.694 | -3.145 | 1.822 | 0.084 | 0.000 |

BCVA, best corrected visual acuity; SE, standard error; n.a., not applicable.
