# Supplementary material for: Network meta-analysis of intravitreal conbercept as an adjuvant to vitrectomy for proliferative diabetic retinopathy
Source: Front Endocrinol (Lausanne). 2023 Feb 22;14:1098165. doi: 10.3389/fendo.2023.1098165 (PMC9989469; doi:10.3389/fendo.2023.1098165)
Supplement: Supplementary file 7 [file Table_4.docx]

**Supplementary Table 4.** Loop-closed inconsistency assessment.

| Loop | IF | SE | z | p | 95% CI | tau2 |
| --- | --- | --- | --- | --- | --- | --- |
| BCVA | | | | | | |
| A-D-F | 0.802 | 0.153 | 5.233 | 0 | (0.50, 1.10) | 0.001 |
| A-D-E | 0.204 | 0.092 | 2.219 | 0.027 | (0.02, 0.39) | 0 |
| D-F-G | n.a. | n.a. | n.a. | n.a. | n.a. | n.a. |
| Operation time | | | | | | |
| A-D-F | 16.017 | 19.009 | 0.843 | 0.399 | (0.00, 53.27) | 152.783 |
| A-D-E | 13.153 | 11.248 | 1.169 | 0.242 | (0.00, 35.20) | 61.423 |
| D-F-G | n.a. | n.a. | n.a. | n.a. | n.a. | n.a. |
| Iatrogenic retinal breaks | | | | | | |
| A-C-D | 0.878 | 1.377 | 0.638 | 0.524 | (0.00, 3.58) | 0 |
| Vitreous hemorrhage | | | | | | |
| A-C-F | 1.557 | 1.329 | 1.172 | 0.241 | (0.00, 4.16) | 0 |
| A-C-D | 0.284 | 1.347 | 0.211 | 0.833 | (0.00, 2.92) | 0 |

BCVA, best corrected visual acuity; IF, inconsistency factor; SE, standard error; CI, confidence interval; n.a., not applicable.
